# Automated measurement of endometrial peristalsis in cine transvaginal ultrasound images

**DOI:** 10.3389/fphys.2022.983177

**Published:** 2022-09-16

**Authors:** Yue Wang, Xiaokun Li, Niya Wei, Yuanxi Liu, Xinting Liu, Ruijie Sun, Chan Huang, Bin Yao, Huifang Wang

**Affiliations:** ^1^ Department of Ultrasound, Peking University Shenzhen Hospital, Shenzhen, China; ^2^ Shenzhen Wisonic Medical Technology Co., Ltd., Shenzhen, China

**Keywords:** endometrial peristalsis, automated analysis algorithm, *in vitro* fertilization and embryo transfer, optical flow technology, transvaginal ultrasonography

## Abstract

**Objectives:** Endometrial peristalsis (EP) in non-pregnant uterine can be assessed by visual assessment of transvaginal ultrasound (TVUS). However, visual assessment is subjective, and the outcome depends on the sonographers and video analysts. This study aimed to create a newly developed automatic analysis algorithm for measuring the EP compared to visual assessment.

**Methods:** A retrospective analysis was performed using the datasets from *in vitro* fertilization and embryo transfer (IVF-ET), who underwent the evaluation of EP by TVUS within 5 days prior to transplantation. 158 cine TVUS images were used to develop the automated analysis algorithm, and 37 cine TVUS images were evaluated by both visual and automated analysis algorithms. The algorithm was developed by applying the optical flow technology and enabled objective analysis of the number, direction, and intensity of EP.

**Results:** The number of peristaltic waves counted by visual assessment was 4.2 ± 2.3 (mean ± standard deviation) and 4.1 ± 2.1 for doctors one and two, respectively. The number of waves counted with the algorithm was 3.6 ± 2.1 at first evaluation and 3.7 ± 2.0 at repeated evaluation. A significant difference was found between the algorithm count and visual assessment (*p* = 0.001, 0.002, 0.003, 0.008). The ICC values for algorithm versus manuals ranged from 0.84 to 0.96 and 0.87 to 0.96. The numbers of the cervix-to-fundus (CF), fundus-to-cervix (FC), and both cervix-to-fundal and fundus-to-cervix (CF + FC) directions of EP counted by the algorithm were 50, 52, and 32, respectively. The numbers counted by visual assessment were 43, 45, and 46, respectively. The number of EP was the same in 87% of the two algorithm counts. The number was lower between the algorithm and visual analysis (79% with complete agreement). The EP intensity assessed by the algorithm was 2.6 ± 1.1, and the peristalsis velocity was 0.147 (0.07) mm/s.

**Conclusion:** The fully automated analysis algorithm can be used to quantify uterine peristalsis comparable to visual assessment.

## 1 Introduction

Endometrial peristalsis (EP) is a stripping movement of the endometrium caused by subtle, wave-like contractions of the sub-endometrial myometrium (Pinto et al., 2015). Peristaltic frequency, direction, and intensity vary according to the menstrual cycle phases under hormonal variations (Wray et al., 2014; Young, 2016). EP plays essential roles in sperm transportation, menstrual discharge, and embryo implantation, which favor pregnancy and the early development of embryos. It has been reported that any change in the velocity and direction of EP compared to its typical characteristics may lead to infertility or pregnancy failure (Kuijsters et al., 2017; Soares et al., 2019). Various diagnostic technologies, such as intrauterine pressure measurement (IUPs), magnetic resonance imaging (MRI), and transvaginal ultrasonography (TVUS), have been introduced to investigate the EP. MRI is costly and not readily available. In the case of IUPs, a significant drawback is that the irritation induced by an intrauterine device may interfere with physiological contraction characteristics, which causes discomfort for patients and makes routine use impractical (Kuijsters et al., 2017; Liu et al., 2018). TVUS, considered a non-invasive, cost-effective, and safe approach for measuring EP, is currently the most appealing method for evaluating EP (Huang et al., 2018; Kuijsters et al., 2019). However, visual assessment is subjective, and the outcome depends on the sonographers and video analysts. The necessary knowledge and skills of a doctor, and thus the need for training and appropriate qualifications (not routinely held at the basic stage of education), are necessary for assessing EP. In addition, the observation and interpretation of EP were too time-consuming to be used in daily practice, even for experienced sonographers (Kuijsters et al., 2019). To overcome these disadvantages, automated analysis of EP in TVUS videos could be a solution. However, the EP is slow and sporadic, different from those shown by cardiac contractility, is regular and distinct, and is not easily assessed automatically. This study aimed to evaluate EP by an automated technique that enables objective analysis using a newly developed automatic analysis algorithm based on optical flow technology and then to compare these results with those from traditional visual assessment by TVUS findings.

## 2 Materials and methods

### 2.1 Study population

A retrospective analysis was performed of 267 patients who underwent *in vitro* fertilization and embryo transfer (IVF-ET) in the reproductive medical center of Peking University Shenzhen hospital between October 2020 and December 2021. Within 5 days prior to transplantation, all patients underwent the evaluation of EP by TVUS. None of the patients had received anticholinergic medications and anti-spasticity agents. The exclusion criteria were the women with uterine pathologies such as adenomyosis, uterine anomaly, uterine fibroids, and polys. Women with intrauterine devices were also excluded. Finally, a total of 195 patients were included in the study. Recorded cine ultrasound images were extracted from a picture archiving and communication system (PACS). Of the 195 cine TVUS images extracted, 158 were used to develop the automated analysis algorithm, and 37 were evaluated by both visual and automated analysis algorithms. Ethical approval was given by the Ethics Committee of Peking University Shenzhen Hospital (No. 2022002). A waiver of informed consent has been obtained for this retrospective study.

### 2.2 Cine transvaginal ultrasound images acquisition

Two ultrasound machines available at our outpatient clinic were used to acquire the cine TVUS images: Resona7 (Mindray Medical Systems, Shenzhen, China), with a 2–9 MHz endovaginal volume transducer (DE10-3WU); and Voluson E8 (GE Healthcare, United States), with a 5–9 MHz endovaginal volume transducer (RIC 5-9-D). These systems had a built-in video record, and the recorded file was later digitized into an AVI/MP4 file.

A standardized scanning protocol was set up, and all the scans were performed according to the following protocol: 1) Let the patient lie in a supine position, keep the body still, and breathe normally. Any artifacts due to respiratory or intestinal movement were excluded; 2) Find the section of the uterus with the largest longitudinal section and the operator holding the probe steady; 3) Collect video data for 2 min; 4) Visual inspection of these ultrasound recordings, replayed at two times the regular speed, was independently performed by two doctors with more than 5 years of TVUS experience and 1 year of experience evaluating EP; 5) When peristalsis occurred, the algorithm and visual assessment evaluated the number and direction of peristalsis. The algorithm only evaluated the intensity and velocity of EP. The number is EP’s number in a time, and the direction of EP was defined by the line connecting the cervix to the fundus. The direction of peristaltic waves was classified as cervix-to-fundus (CF), fundus-to-cervix (FC), and both cervix-to-fundal and fundus-to-cervix (CF + FC). The EP’s velocity is defined as the time it takes one peristalsis wave from the beginning to the end. The velocity is the length of a path (mm) divided by the time (s) it takes for the peristalsis to complete the path. The EP’s intensity is to calculate each peak on the peristaltic wave curve and generate a point with a peristaltic range on the x-axis and peristaltic amplitude on the y-axis in the coordinate system.

### 2.3 Development of automated analysis algorithm

The algorithm had three main processing stages: inputting cine images for feature extraction, model establishment, and evaluation.

#### 2.3.1 Algorithm establishment

##### 2.3.1.1 Motion capturing

**FIGURE 1:**
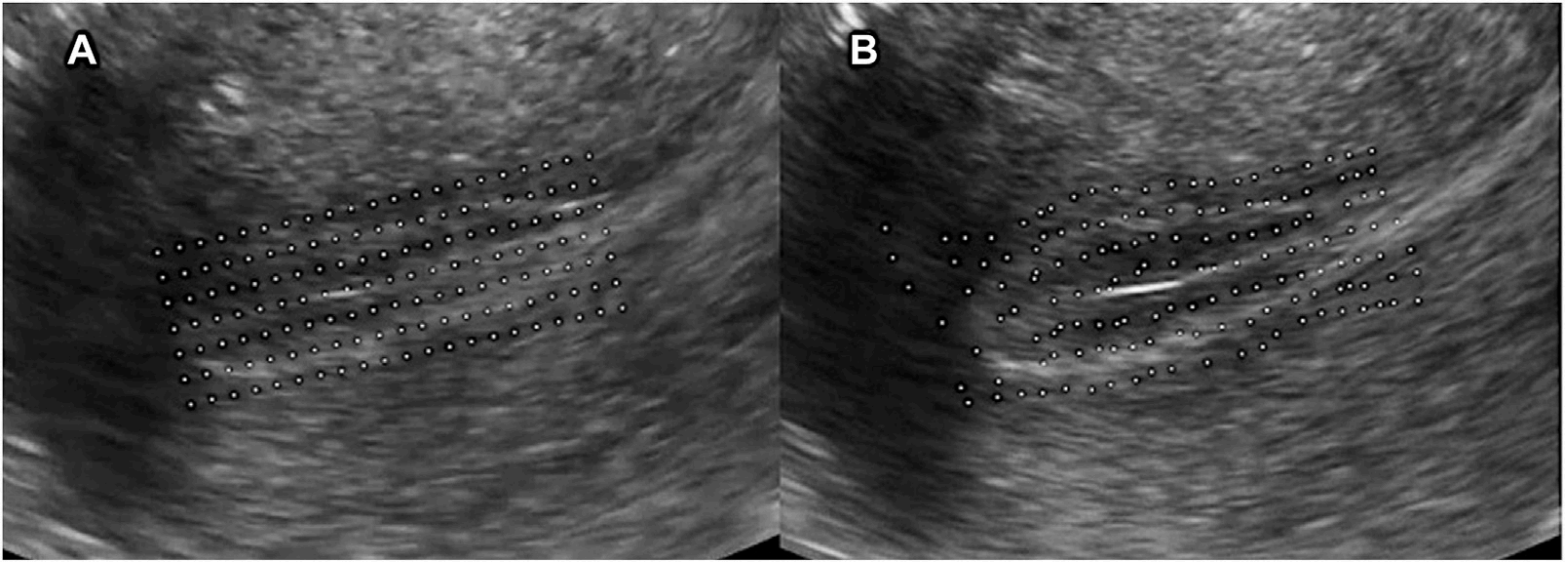
**(A)** Feature points generation. First, determined the rectangle that encircles the endometrium area and filled this rectangle area with aligned feature points. The feature points are equally spaced, and the interval is usually 15-pixel-length; **(B)** Displacement of feature points.

**FIGURE 2:**
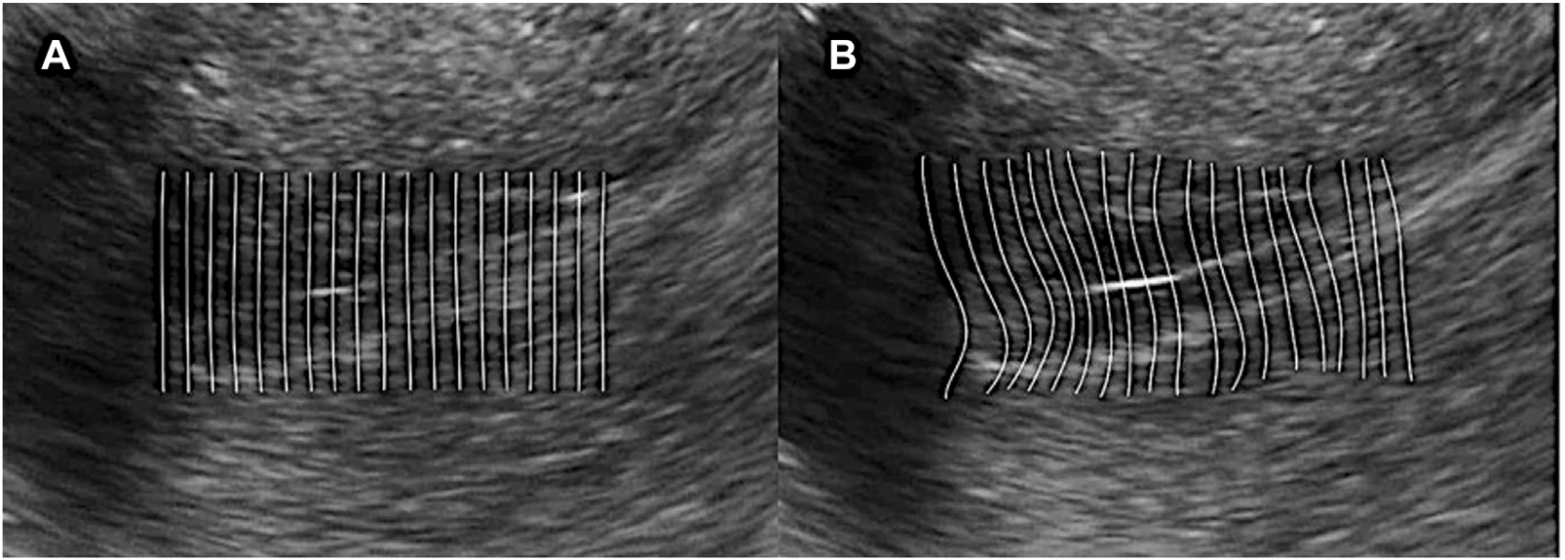
Computing feature value. **(A)** Regard points in each row as a line and fit points whose initial position is in the same row with a straight line; **(B)** Peristalsis deforms the straight line into a curve. The line curves in the same direction as peristalsis, and the curvature is in proportion to the magnitude of peristalsis.

1) Feature points generation: when the video was imported, the rectangle was determined that encircle the endometrium area and fill this rectangle area with aligned feature points. The feature points are equally spaced, and the interval is usually 15-pixel-length. The initial coordinates of each feature point are recorded then (Figure 1A).
2) Displacement of feature points: the unit time “t” was assumed as the time duration of two adjacent video frames. Then the velocity of each feature point is simplified to its displacement between two adjacent frames, Δx . Furthermore, the new coordinates can be expressed as $x_{t}=x_{0}+\Delta x$ (Figure 1B).
3) Temporal and spatial filtering: Due to the background noise of ultrasound, the gray value of the picture is constantly changing, which can cause an error during calculation. After coordinate data were collected from each frame, the motion information could not be obtained from this data directly. Instead, temporal and spatial smoothing processing was needed first.
4) Computing feature value: Since the peristalsis of the endometrium is a continuous motion, which has spatial continuity. It means the displace of each single feature point will not describe the whole motion and should consider this question from a macro viewpoint. If we regard points in each row as a line, we fit points whose initial position is in the same row with a straight line. And then, the peristalsis will deform the straight line into a curve. If we compare the left and right sides of Figure 2, one can observe that the line curves in the same direction with peristalsis, and the curvature is in proportion to the magnitude of peristalsis. In this condition, we can say that the curvature of those fitting curves can present the magnitude of endometrium peristalsis. We use the variation of curvature of two frames instead of curvature from a single frame since it is static. So we calculate the difference by subtracting the curvature in the frame that is five frames ahead of the curvature in the current frame, and the variation is Δτ . (If there are less than five frames ahead of the current frame, then subtract with the first frame). In addition, we prefer to record this variation data in two parts: sign and absolute value. The absolute value presents the magnitude, and the sign stands for direction, which we will discuss later.

##### 2.3.1.2 Motion amplifying

1) Salient motion determinant: we define the absolute value of curvature variation as the peristalsis magnitude parameter. Since the numerical value of magnitude from samples is different, we need to normalize the curvature variation data obtained from the last step to make it easier to determine the magnitude level. We use a linear normalization function that can map all the magnitude data into sections [0, 1] uniformly:

$$y_{0}=\phi \left(\left|\Delta \tau \right|\right)\in \left[0,\;1\right].$$ (1)

And then, we can define a threshold value α , for a value $y_{0}$ larger than α can be regarded as salient peristalsis. The one less than α will be regarded as non-peristalsis. (The threshold value α can be modified from 0 to 1, in this experiment, we choose α=0.6) :

$$y_{1}=y_{0}-\alpha .$$ (2)

2) Rendering weight parameter: We wish to visualize the peristalsis in a color rendering way: the more salient the peristalsis is, the brighter the peristalsis area will be (High rendering weight); and vice-versa, the area without peristalsis will not be rendered (Low rendering weight). We modify the weight parameter by the Sigmoid function:

$$y_{2}=sigmoid\left(y_{1},\beta \right)=\frac{1}{1+e^{-\beta \cdot y_{1}}}.$$ (3)

β controls the slope of the Sigmoid function, a larger slope means the peristalsis area will have higher weight, and the non-peristalsis area will get lower weight.

**FIGURE 3:**
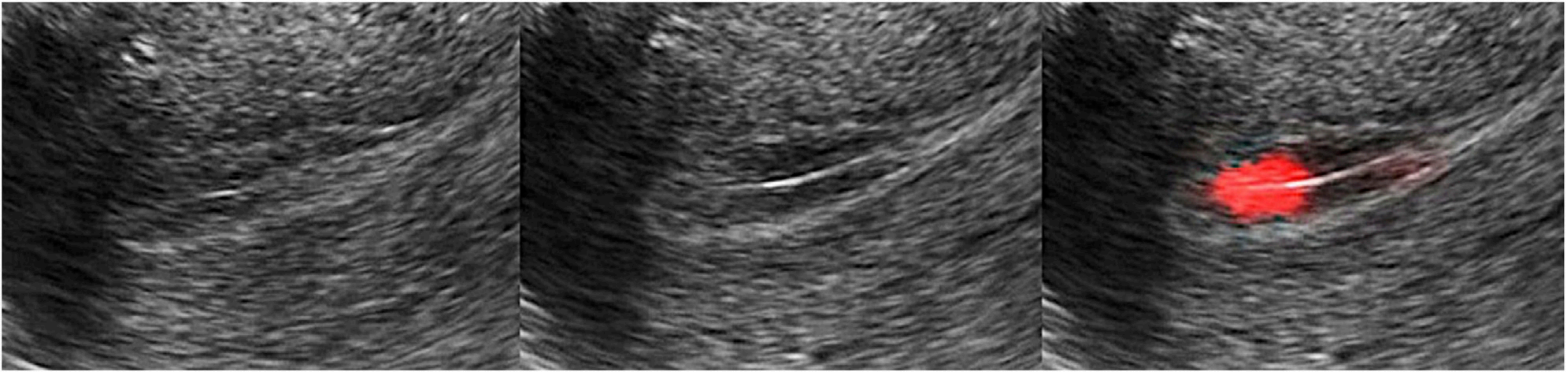
Rendering display. Multiply the color value with the rendering weight and add the value into the RGB channel of the original frame. The area with salient peristalsis was bright red, and no peristalsis kept the same grey value.

3) Rendering display: To display the final rendering results, we multiply the color value with the rendering weight and add them into the RGB channel of the original frame. We also need interpolation to the whole endometrium area since we only have the value in feature points (Figure 3). As can be seen, the area with salient peristalsis is bright red. On the contrary, the area with no peristalsis keeps the same grey value.
4) Motion graph generation: Since the diversity of the endometrium orientation in different samples, we define the left orientation (for horizontal position) and downward orientation (for vertical position) as “forward direction”; The right orientation and upward orientation correspond for “backward direction.” Remember, we have recorded the sign of Δτ , which presents the peristalsis direction: “+1” stands for “forward direction” and “−1” stands for “backward direction.”

**FIGURE 4:**
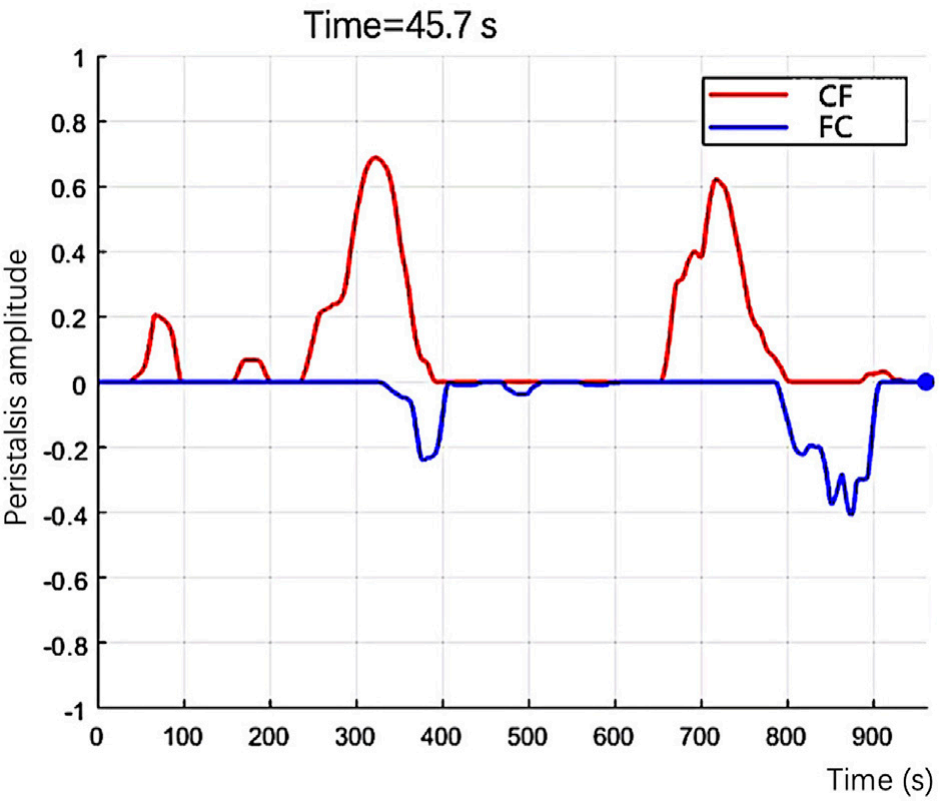
In the graph, the number of the feature points presents the size of the peristalsis area, and the curve’s color presents the direction of the peristalsis.

We only concern the salient peristalsis area, the feature points with amplitude parameters larger than α . We count the number of those points of ‘forward direction’ and ‘backward direction’ separately and then label them as “n_+” and “n_−.” We plot “n_+” and “n_−” on the Cartesian coordinate, then obtain the motion graph. In the graph, the number of the feature points presents the size of the peristalsis area, and the curve’s color presents the direction of the peristalsis (Figure 4). It is easy to observe some features of peristalsis with different modes:

A) In a one-way peristalsis motion graph, the curve presenting the “forward direction” (or “backward direction”) is always above the other one, and the curve of the opposite direction will always keep zero value.
B) For the peristalsis that first move “forward” and then move “backward” (or first “backward” then “forward”), its graph has the feature that the “peak” of the curve will appear alternately.
C) If the “forward” and “backward” peristalsis happen simultaneously, then their “peak” of curves will also appear simultaneously in the graph.

#### 2.3.2 Algorithm evaluation

**FIGURE 5:**
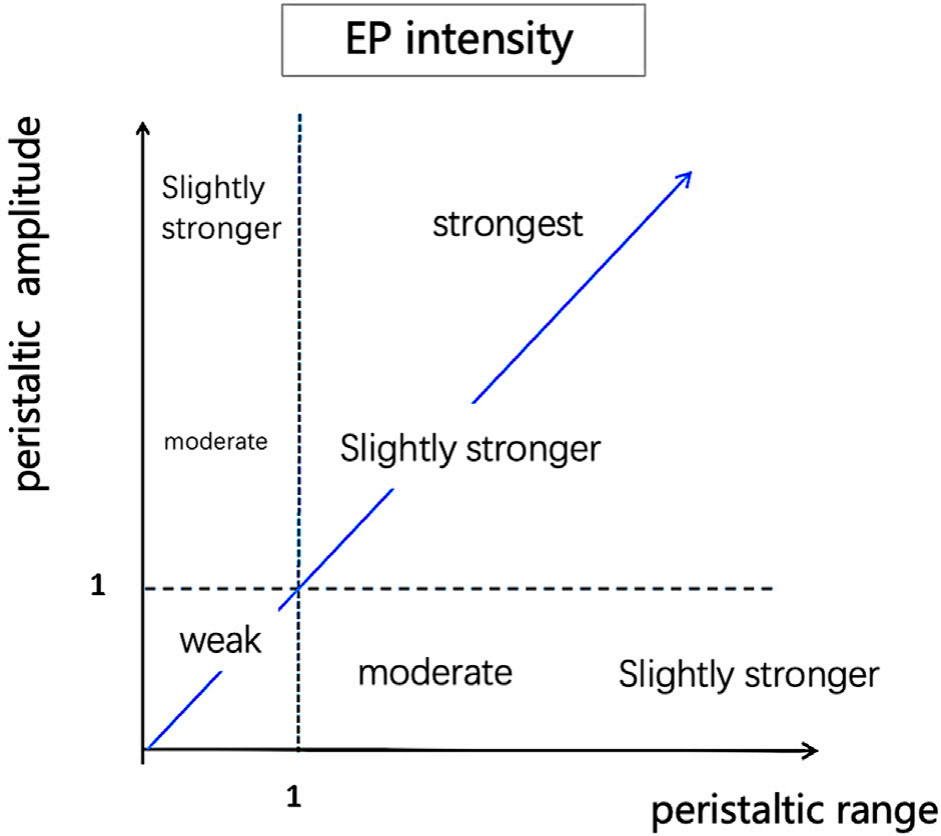
Quantitative assessment of EP intensity. Calculate each peak on the peristaltic wave curve and generate a point with peristaltic range on the x-axis and peristaltic amplitude on the y-axis in the coordinate system.

**FIGURE 6:**
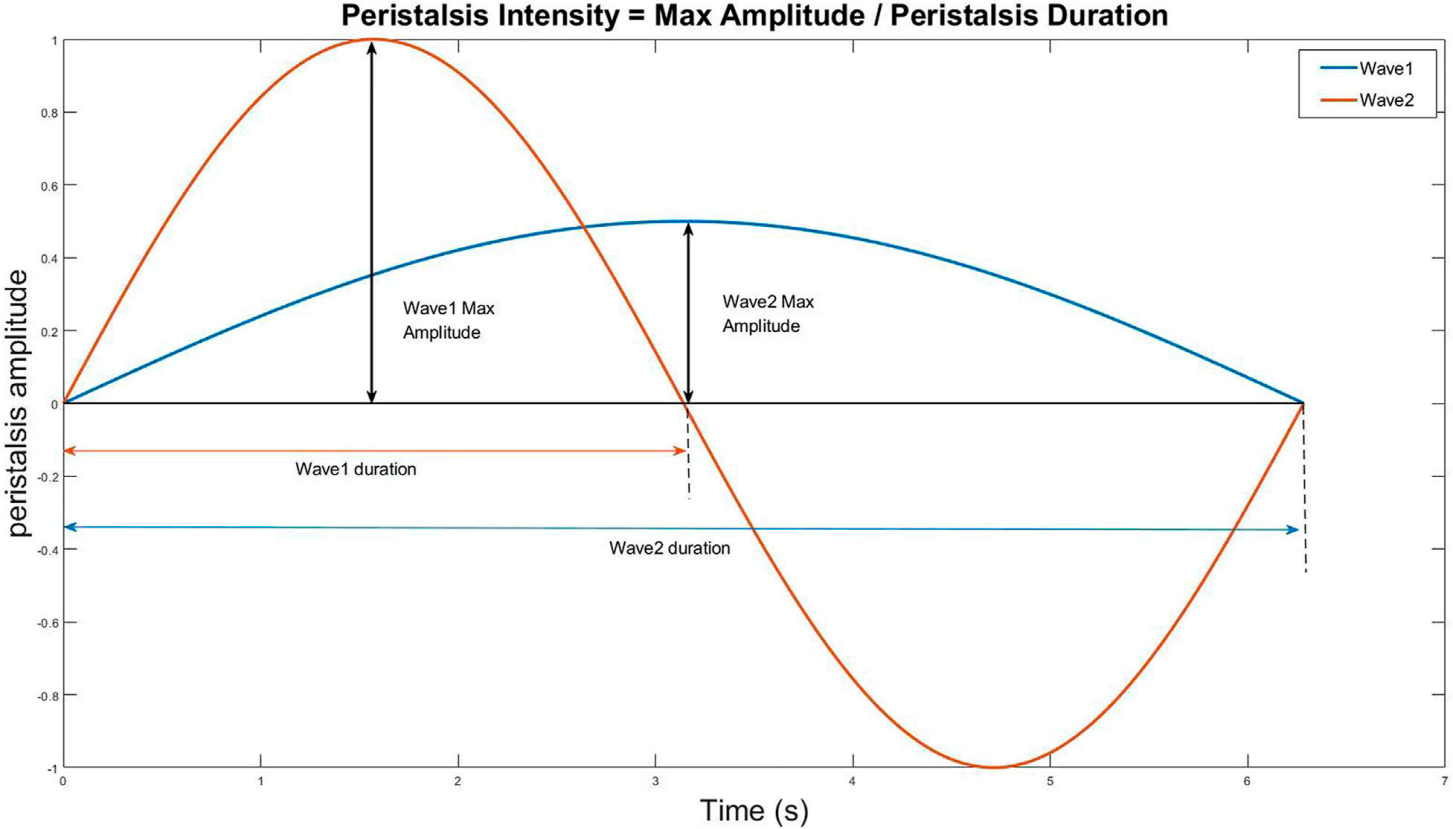
The ratio of wave peak to wavelength (i.e., the ratio of ordinate to abscissa) as the indicator of peristalsis velocity.

(1) The established algorithm evaluated EP’s number in 37 cine ultrasound datasets twice, and two physicians also evaluated the number. The repeatability of the algorithm evaluation and the agreement between the algorithm and the visual assessment was calculated.
(2) Researchers extracted 134 cine ultrasound images containing only one EP from 37 datasets. The algorithm and two sonographers evaluated EP’s direction in 134 cine ultrasound images simultaneously. The consistency of EP’s direction evaluation between the algorithm and the visual assessment was calculated.
(3) Quantitative assessment of EP intensity and velocity
  1) The intensity of EP was calculated at each peak on the peristaltic wave curve and generated a point with a peristaltic range on the x-axis and peristaltic amplitude on the y-axis in the coordinate system (Figure 5). The classification of EP’s intensity is defined according to the following criteria:
    A) weak: the peristalsis wave range and peristalsis amplitude are both less than 1;
    B) moderate: either peristalsis wave range or peristalsis amplitude is greater than 1;
    C) strong: both the peristalsis wave range and amplitude are greater than 1;
  2) Even if the peristaltic range is the same, some waves are fast while others are slow. We use the ratio of wave peak to wavelength (i.e., the ratio of ordinate to abscissa) as the indicator of peristalsis velocity (Figure 6).

### 2.4 Statistical analysis

Continuous data were expressed as mean plus/minus standard deviation, and normal distribution was tested using the Shapiro-Wilk test. The EP’s numbers counted by the algorithm and visual assessment were compared with the Wilcoxon signed-rank test. The concordance was computed using the intraclass coefficient correlation (ICC). The repeatability of two algorithm counts for one video was evaluated using ICC; ICC evaluated the agreement between algorithm and visual analysis; ICC evaluated the inter-reader agreement between two visual assessments. ICC of less than 0.20 denotes poor repeatability, 0.21–0.40 fair, 0.41–0.60 moderate, 0.61–0.80 good, and 0.81–1.00 excellent repeatability (Youssef et al., 2016). All statistical analyses were performed using SPSS Statistics 26 (IBM SPSS Statistics for Mac, Version 26.0).

## 3 Results

1. In Supplementary Video S1 was an example of the automated analysis algorithm that evaluated EP. The red lines were the peristalsis from the cervix to the fundus, and the blue lines were the fundus to the cervix. The video shows three peristalsis waves from the cervix to the fundus and two from the fundus to the cervix.
2. Repeatability and consistency of EP’s number counts

**FIGURE 7:**
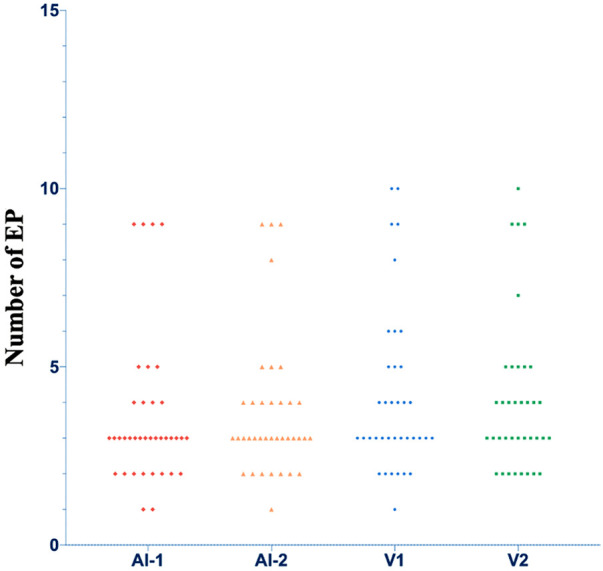
The distributions of the number of EP. AI, Artificial intelligence algorithm; V, visual analysis.

The algorithm and visual assessment analyzed a total of 37 cine ultrasound images. The datasets of EP’s number did not conform to a normal distribution according to a Shapiro-Wilk test (*p* = 0.000). The mean (and standard deviation) of the EP’s number counted by visual assessment was 4.2 ± 2.3 and 4.1 ± 2.2 for doctors one and two, respectively. The EP’s number counted with the algorithm was 3.6 ± 2.1 at first evaluation and 3.7 ± 2.0 at repeated evaluation. A significant difference was found between the algorithm counts and visual assessments (*p* = 0.001, 0.002, 0.003, 0.008). The distributions of the EP’s number are shown in Figure 7.

The repeatability of the two algorithm counts was excellent, with an ICC value of 0.97 (*p* = 0.000). The number of five datasets was different; among them, three datasets had a difference of 1 peristalsis wave, and two datasets had a difference of 2 peristalsis waves. The algorithm counts also showed excellent agreement with the visual assessment of docotor1 (ICC values ranging from 0.84 to 0.96, *p* = 0.000) and doctor 2 (ICC values ranging from 0.87 to 0.96, *p* = 0.000). The inter-reader agreement between doctors 1 and 2 was excellent (ICC values ranging from 0.92 to 0.98, *p* = 0.000). ICCs value is summarized in Table 1.

3. EP direction assessment

**TABLE 1:** Agreement in number of endometrial peristaltic waves counted by different methods.

Methods	Mean (and standard deviation)	ICC
Algorithm evaluation 1st	3.6 (2.1)	
Algorithm evaluation 2nd	3.7 (2.0)	
Visual reader 1	4.2 (2.3)	
Visual reader 2	4.1 (2.2)	
Algorithm evaluation 1st vs.2nd		0.97
Visual reader 1 vs. 2		0.96
Algorithm evaluation 1st vs. visual reader 1		0.91
Algorithm evaluation 1st vs. visual reader 2		0.93
Algorithm evaluation 2nd vs. visual reader 1		0.91
Algorithm evaluation 2nd vs. visual reader 2		0.94

A total of 134 cine ultrasound images containing only one EP were analyzed by algorithm evaluation and visual assessment. The number of cine ultrasound images with the direction of EP classified into CF, FC, and CF + FC per method is shown in Table 2. Mixed CF + FC direction was observed in 24% of cine ultrasound images by algorithm, while this pattern was slightly less frequent in visual assessment (22%). In the algorithm, the number of same-direction EP was the same in 87% of the two algorithm counts. The ratio was lower between the algorithm evaluation and visual assessment (79%) and between the two sonographers (66%) (Table 2).

4. The mean EP intensity assessed by the algorithm was 2.6 ± 1.1, and the mean peristalsis velocity was 0.147 (0.07) mm/s.

**TABLE 2:** Direction of endometrial peristaltic waves evaluated by different methods.

	The same number	Percentage in total (%)
Algorithm evaluation 1st vs. 2nd	116	0.87
Algorithm evaluation 1st vs. visual reader 1	106	0.79
Algorithm evaluation 1st vs. visual reader 2	97	0.72
Algorithm evaluation 2nd vs. visual reader 1	97	0.72
Algorithm evaluation 2nd vs. visual reader 2	88	0.65
Visual reader 1st vs. 2nd	89	0.66

## 4 Discussion

The developed algorithm could automatically measure the number, direction, velocity, and intensity of EP in cine ultrasound images. The results indicated that the algorithm is reliable, objective, and reproducible for measuring EP. The study demonstrated that the algorithm had good repeatability in evaluating EP’s number. The number was precisely the same between the two evaluations in 32 out of 37 cine ultrasound images, and the remaining five showed only one or two differences in the repeated evaluation. The results also showed that the algorithm evaluation was in close agreement with the visual assessment. The number recognized by the algorithm was less than that recognized by visual assessment (3.6 vs. 4.2), and the difference is statistically significant. By analyzing the cine ultrasound images, it was found that most differences occurred in the video with the CF + FC peristalsis wave. The possible reason might be that the sensitivity of vision to time resolution is inferior to the algorithm. The visual evaluated CF + FC as two EPs while the algorithm as one. The algorithm evaluation of EP direction was consistent with the visual assessment. In addition to the common CF, FC, and CF + FC, the EP direction also has the following conditions: 1) Peristalsis starts in the middle of the uterine corpus and then peristalsis to the uterine fundus and cervix at the same time; 2) Peristalsis co-occurs in different directions at multiple starting points; 3) The direction of peristalsis is inconsistent with the longitudinal axis of the uterus, showing irregular peristalsis. The above conditions lead to difficulty in judgment by visual assessment and algorithm evaluation. In this study, there were no datasets with 0 number of EP by visual assessment, so it was impossible to judge the advantage of the algorithm over visual assessment in spatial resolution.

This study has two noteworthy strengths. First, an assessment based on a multi-indicator approach could provide more comprehensive information for the clinical practice. Not only the number and direction but also the velocity and intensity of EP could be evaluated by the algorithm. Peristaltic waves of the same number and direction must have different physiological and physical effects on the endometrium if the peristaltic range is too extensive or the velocity is too fast. No published studies have assessed the intensity and velocity of peristalsis by ultrasound. Second, EP’s four indicators are presented in coordinates, the number of peristalses was the number of waves, the direction was different colors up and down the X-axis, and the amplitude of the wave displays the intensity of the peristalsis. The velocity of peristalsis is the wave’s speed in the video’s coordinate system.

Although EP has been extensively studied as a factor affecting fertility since the 1990s, the assessment is currently not used as a routine examination in clinical practice, mainly due to the lack of an efficient, objective, accurate assessment method. IUPs are theoretically the most accurate and objective for determining all effects and dimensions of EP. A significant drawback of IUPs is that the device causes the patient discomfort. In addition, irritation induced by an intrauterine device may interfere with physiological contraction characteristics (Wray et al., 2014). MRI can measure the frequency of EP but not amplitude. MRI has a higher detection rate because it is more advantageous in displaying sub-endometrial wave conduction. However, MRI is expensive and time-consuming (Kido et al., 2011; Nakai et al., 2012; Watanabe et al., 2014). Tasnim’s team (Tasnim et al., 2019) and Watanabe’s team (Watanabe et al., 2014) investigated the number of EPs automatically assessed by MRI imaging.

Van Gestel et al. (2007) used ultrasound to evaluate EP. The study showed that the interobserver agreement among the three investigators resulted in a kappa value of 0.83, reflecting strong agreement. The study did not explore the consistency of contraction amplitude. Mori’s team established a model for predicting pregnancy outcomes by ultrasound assessment of uterine motion velocity (Morizaki et al., 1989). Huang’s team applied speckle tracking technology to automatically assess the velocity and direction of contraction waves (Huang et al., 2018).

Limitations of our study include its retrospective nature and the small sample size. EP was only analyzed from a methodological point of view and was not evaluated in conjunction with clinical pregnancy outcomes. Future prospective studies of EP combined with clinical pregnancy outcomes and different menstrual cycles are needed. As the algorithm is in the experimental stage, technical problems such as complex programs and motion artifacts will be solved in the future.

In conclusion, we developed the automated analysis algorithm based on optical flow technology, which can comprehensively evaluate EP’s number, direction, intensity, and velocity in cine ultrasound images. The algorithm can improve the efficiency of clinical evaluation of EP and has potential application prospects.

## Data Availability

The original contributions presented in the study are included in the article/Supplementary Material, further inquiries can be directed to the corresponding author.

## Supplementary material

SUPPLEMENTARY VIDEO S1 An example of the automated analysis algorithm evaluated EP. The red lines were the peristalsis from the cervix to the fundus, and the blue lines were the fundus to the cervix. The video shows three peristalsis waves from the cervix to the fundus and two from the fundus to the cervix.
